# *In Situ* Immunofluorescence Imaging of Vital Human Pancreatic Tissue Using Fiber-Optic Microscopy

**DOI:** 10.1155/2024/1397875

**Published:** 2024-06-06

**Authors:** Sophia Ackermann, Maximilian Herold, Vincent Rohrbacher, Michael Schäfer, Marcell Tóth, Stefan Thomann, Thilo Hackert, Eduard Ryschich

**Affiliations:** ^1^ Department of Surgery University of Heidelberg, Heidelberg, Germany; ^2^ Department of Pathology University of Heidelberg, Heidelberg, Germany

## Abstract

**Purpose:**

Surgical resection is the only curative option for pancreatic carcinoma, but disease-free and overall survival times after surgery are limited due to early tumor recurrence, most often originating from local microscopic tumor residues (R1 resection). The intraoperative identification of microscopic tumor residues within the resection margin *in situ* could improve surgical performance. The aim of this study was to evaluate the effectiveness of fiber-optic microscopy for detecting microscopic residues in vital pancreatic cancer tissues. *Experimental Design*. Fresh whole-mount human pancreatic tissues, histological tissue slides, cell culture, and chorioallantoic membrane xenografts were analyzed. Specimens were stained with selected fluorophore-conjugated antibodies and studied using conventional wide-field and self-designed multicolor fiber-optic fluorescence microscopy instruments.

**Results:**

Whole-mount vital human tissues and xenografts were stained and imaged using an *in situ* immunofluorescence protocol. Fiber-optic microscopy enabled the detection of epitope-based fluorescence in vital whole-mount tissue using fluorophore-conjugated antibodies and enabled visualization of microvascular, epithelial, and malignant tumor cells. Among the selected antigen-antibody pairs, antibody clones WM59, AY13, and 9C4 were the most promising for fiber-optic imaging in human tissue samples and for endothelial, tumor and epithelial cell detection.

**Conclusions:**

Fresh dissected whole-mount tissue can be stained using direct exposure to selected antibody clones. Several antibody clones were identified that provided excellent immunofluorescence imaging of labeled structures, such as endothelial, epithelial, or EGFR-expressing cells. The combination of *in situ* immunofluorescence staining and fiber-optic microscopy visualizes structures in vital tissues and could be proposed as an useful tool for the *in situ* identification of residual tumor mass in patients with a high operative risk for incomplete resection.

## 1. Background

Pancreatic cancer is an aggressive malignancy with a poor prognosis [1]. Standardized therapies, such as chemotherapy and radiotherapy, have failed to cure this cancer in a great majority of patients. Complete surgical resection remains the only curative treatment for pancreatic cancer [1]. The mean 5-year survival rate of patients is less than 10% [2]. Therefore, there is an urgent need to develop effective therapeutic strategies for the treatment of pancreatic cancer.

Complete tumor resection in pancreatic cancer is a well-standardized but technically demanding surgical procedure [1]. Its primary aim is complete removal of the tumor-bearing part of the pancreas and the surrounding tissues, such as the duodenum, gallbladder, pylorus, and lymphatic nodes. The extent of the operation depends on the tumor size and location, which can be determined preoperatively using computed tomography or other cross-sectional imaging modalities [3]. However, the exact extent of tumor infiltration cannot be determined using these techniques. Although the surgeon's aim is complete resection (R0), the diffuse and aggressive growth pattern and extensive perineural invasion complicate complete resection, so undetected microscopic tumor residues may remain in the patient's body, mainly at the resection margins (R1 resection) [4, 5]. Microscopic tumors can be identified intraoperatively using frozen sections or postoperatively by histological analysis of the dissection margin in resected specimens by pathologists [6–8]. The proportion of R1-resected patients is high and can reach up to 76% using a standard pathological protocol [6]. The most recent clinical studies show that R1 resection results in shorter disease-free and overall survival than R0 resection, which underlines the importance of microscopically complete resection [7–10].

Anatomically, pancreatic resection margins can be divided into several parts [11], with the posterior margin most frequently observed to contain microscopic tumor residues [6, 8]. The “gold standard” for intraoperative detection of microscopic tumor residues in pancreatic cancer is the use of intraoperative frozen sections [4, 5], which is a routine procedure in our department. However, only a small part of the resection margin, usually the pancreatic neck, is routinely analyzed using this procedure. Moreover, pathological analysis of the entire resected specimen along all margins is time-consuming and can only be performed after surgery.

In recent years, molecular imaging using fluorescence-labeled antibodies has been suggested for intraoperative detection of pancreatic cancer [12]. This technique is based on near-infrared macroimaging after the systemic injection of fluorescence-labeled antibodies and can be combined with photoacoustic techniques [13, 14]. A clinical pilot study reported that this technique is safe for use in patients. Moreover, the feasibility of this technique has only been demonstrated at the macroscopic level [13, 14].

Fiber-optic microscopy (FOM) is an innovative technique in which light is transmitted to an object using an optical fiber or fiber bundle [15, 16]. The method can be based on either wide-field or laser scanning confocal microscopy and is well suited for endomicroscopic applications. It is used clinically for microscopic identification (optical biopsy) of superficial lesions of the gastrointestinal tract [17–19], pseudocysts [20], and needle biopsies [21].

Recently, we constructed a compact FOM system that translates the microscopic image through a thin Ø 0.16-0.85 mm fiber bundle of variable length. The fiber bundle can be placed on or inserted into the tissue and provides high-quality microscopic fluorescence imaging. As previously demonstrated, the system enables excellent visualization of tissue morphology at the microscopic level, such as microscopic blood vessels labeled after topical application of fluorescent antibodies in mice [22]. Our self-designed wide-field system has a substantially higher sensitivity than available fiber-optic laser scanning confocal systems [22].

It was proposed that FOM could be useful for rapid intraoperative assessment of tumor residues *in situ* and could be helpful for surgeons to decide whether more extensive resection is required to achieve R0 status. Previous studies on pancreatic cancer in rats [23, 24] and other cancer entities in humans [25] have mainly focused on the feasibility of intraoperative endomicroscopy as a means of supportive diagnostics [25]. The aim of the present work was to study the use of a combination of antibody-based immunofluorescence labeling and FOM to identify vital pancreatic cancer cells and microscopic tumor remnants *in situ*. Epithelial cell-specific (anti-EpCAM, anti-cytokeratin 8, or 18), proliferating cell-specific (anti-CD71), and endothelial cell-specific (anti-CD31) antibodies conjugated to a different set of fluorophores were selected to meet this aim.

## 2. Methods

### 2.1. Tissue and Cell Lines

Pancreatic tissue samples were obtained from the biobank of the European Pancreas Center (Pancobank) at the Department of General and Visceral Surgery of the University Hospital of Heidelberg in accordance with the regulations of tissue banks and the approval of the ethics committee of the University of Heidelberg.

The presence of tumor cells in single tissue specimens was investigated using H&E-stained slides (see below). Snap-frozen and fresh whole-mount tissue specimens were used in this study. Tumor tissue samples are summarized in Table 1(a). In addition, 7 frozen and 12 fresh samples of tumor-distant pancreatic tissue (assigned as “normal pancreas”) were used.

The following human cell lines were used for in vitro experiments: AsPC-1, DAN-G, Fampac, MiaPaCa-2 (CLS Cell Lines Service, Heidelberg, Germany), Pk-9 (Tohoku Medical School, Sendai, Japan), and Patu8988t (DSMZ, Braunschweig, Germany). Cells were grown in Iscove's medium supplemented with 10% fetal calf serum, L-glutamine (2 mM), penicillin (100 U/mL), and streptomycin (100 *μ*g/mL) (all from CCPro, Oberdorla, Germany) at 37°C in an incubator (Bender, Tuttlingen, Germany) in a humidified atmosphere containing 5% carbon dioxide (Guttroff, Wertheim-Reicholzheim, Germany).

### 2.2. Chorioallantoic Membrane Assay and 5-Fluouracil Treatment

Tumor growth in the chorioallantoic membrane (CAM) was assessed as previously described [26]. For tumor inoculation, the tumor cell suspension (approximately 1 × 10^6^ cells) was mixed with Matrigel® (Corning Inc., Corning, NY, USA), and 25 *μ*L of this mixture was inoculated on the chorioallantoic membrane on the 9th day after fertilization. Imaging of vital microscopic tumors was performed two days after inoculation (11th day after fertilization). Local chemotherapy was performed by daily topical application of a 5-fluouracil- (5-FU-) soaked piece of filter paper (7-10 *μ*L, 50 mg/mL; Medac, Wedel, Germany) applied over 3 or 6 days beginning on the second day after inoculation. Local application of phosphate-buffered saline (PBS) using soaked filter paper was used as a control. The tumor labeling with fluorescent antibodies was performed as described below. The number of tumors that were identified at the end of treatment using macroscopic only or combined macroscopic and FOM inspection was counted and expressed as a percentage of the total number of inoculated tumors.

### 2.3. Immunofluorescence Staining

Antigen-specific monoclonal (mAbs) (Table 1(b)) and respective isotypic control antibodies were used for immunofluorescence staining (Table 1(c)). All antibodies except one were purchased from BioLegend (San Diego, CA). Nonlabeled antibodies were conjugated with Alexa Fluor 647 using its NHS ester (Thermo, Rockford, MA, USA) according to the manufacturer's instruction (Thermo). For immunohistochemistry of frozen tissue, 7 *μ*m acetone-fixed slides were stained with DAPI (1 *μ*M; Thermo), Alexa Fluor 647-conjugated WM59 mAb, and PE-conjugated mAb (2 *μ*g/mL; BioLegend) for 30 min at room temperature. The slides were washed with PBS and covered with a cover slip (Paul Marienfeld, Lauda-Königshofen, Germany) and mounting medium (Fluoromount G; Thermo).

For staining of fresh whole-mount tissue, specimens were manually sliced into pieces approx. 2 mm thick. The size of the single specimens allowed us to prepare 2-3 slices to be used for staining with different antibodies. The numbers of whole-mount tissue samples stained with individual antibody clones are shown in Suppl. Table 1. Tissue slices were incubated in PBS containing Hoechst 33342 (4 *μ*M; Thermo) and conjugated antibodies (2 *μ* g/mL) for 30 min. After staining, the slices were washed in PBS, placed between two cover slips (Paul Marienfeld), fixed with viscous silicone paste (Kurt Obermeier, Bad-Berleburg, Germany), and subjected to wide-field fluorescence microscopy (WFM) imaging as described below. FOM was subsequently performed after WFM. For this purpose, the cover glass was removed, and imaging was performed by direct contact between the fiber bundle and the tissue. Cells were stained in vitro using the same procedure as for the histological slides. For FOM imaging, the fiber bundle was carefully placed on the cell monolayer. Careful contact with cells helped to avoid mechanical cell detachment.

To analyze the antibody penetration depth, whole-mount pancreatic tissue was incubated with Alexa Fluor 647-conjugated WM59 or 9C4 antibodies for 30 s or 30 min, cross-sectioned, placed between cover slips, and studied using WFM. For tumor labeling in CAM, the antibody (15 *μ*L, 10 *μ*g/mL) was topically applied using a minibrush, followed by washing with PBS after a short incubation period. Hematoxylin and eosin staining of 7 *μ*m slides was performed using Mayer's hemalum (Merck, Darmstadt, Germany) and Eosin G (Carl Roth, Karlsruhe, Germany).

### 2.4. WFM and FOM Imaging

**Figure 1:**
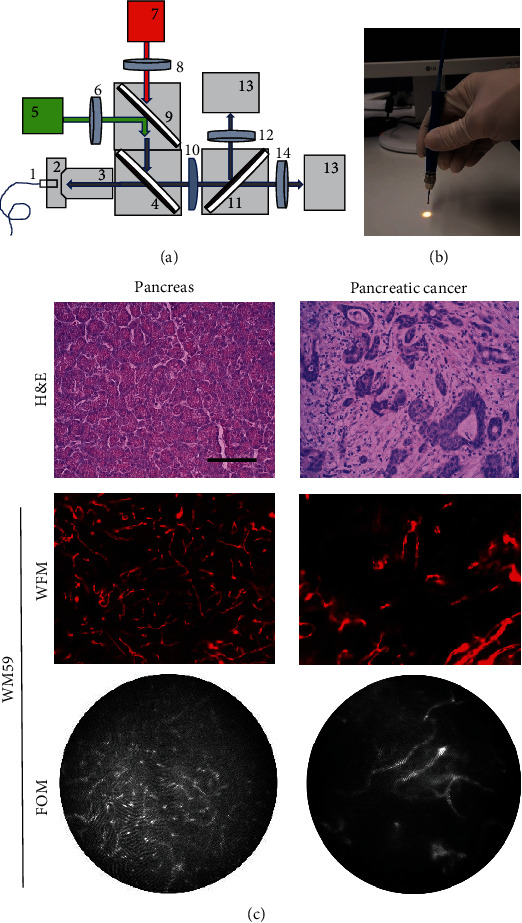
(a) Design of FOM microscopy system: (1) fiber bundle GT-FIGH30-850N, Grintech; (2) high-precision zoom, Thorlabs, Newton, NJ, USA; (3) objective Plan-Neofluar 10x/0,3, Zeiss; (4) multiband beamsplitter 87-287, Edmund Optics, Barrington, NJ, USA; (5) LED MINTL, Thorlabs; (6) bandpass filter 555/30, Zeiss; (7) LED M660L4, Thorlabs; (8) bandpass filter 620/40, Zeiss; (9) beamsplitter DMLP550R, Thorlabs; (10) lens LA1986, Thorlabs; (11) beamsplitter 87-060, Edmund Optics; (12) bandpass filter FB580/10, Thorlabs; (13) camera CD505MU, Thorlabs; and (14) bandpass filter FB680/10, Thorlabs. (b) View of the end piece of fiber optics fixed in the holder. (c) Representative WFM and FOM images of vital whole-mount tissue (pancreas versus pancreatic cancer), stained with anti-CD31 WM59 mAb, PE-conjugated, and respective H&E staining; bar 200 *μ*m, FOM diameter 790 *μ*m.

WFM imaging was performed using a Zeiss Observer Z1 system (Zeiss, Jena, Germany) equipped with Colibri LED sources, filter set 90 HE, and objective (Plan-Neofluar 10×/0.3; Zeiss). The specifications of fluorescence detection using the WFM are listed in Suppl. Table 2. For FOM imaging, a self-designed system was constructed, as shown in Figures 1(a) and 1(b). Detailed information regarding the conjugated fluorophores and the excitation and emission wavelengths using the FOM are summarized in Suppl. Table 3. The flow of light (*φ*_e_) illuminated by the WFM (Suppl. Table 2) or FOM system (Suppl. Table 2) was measured in lx using a luxmeter (Voltcraft MS-200LED, Conrad Electronic, Hirschau, Germany) and calculated in mW/cm^2^ using the following formula [27]: *ϕ*_*e*_ = lux/*k*(*λ*) where *κ*(*λ*) is the coefficient of spectral sensitivity of the human eye [27].

Fluorescence staining of histological slides and whole-mount tissue was quantified by image-based analysis of mean fluorescence intensity (MFI), as previously described [28, 29]. The raw MFI of cells specifically stained with Alexa Fluor 647- or PE-conjugated antibodies was measured and corrected by subtracting the determined background MFI. The MFI of the simultaneously stained microvascular blood vessels was used as a reference. The ratio between the MFI values of these stains was calculated and used as a value independent of staining variation.

To study the sensitivity and specificity of FOM for tumor discrimination, tissues were cut into approximately 1 mm^3^ pieces and stained with the antibodies mentioned above. They were placed in thin nontransparent conical tubes and assigned to “tumor” or “no tumor” by FOM using double-blinded manner.

### 2.5. Identification of Tumor Area Using Hematoxylin-Eosin and Immunofluorescence Staining

After immunofluorescence imaging, all the tissue slices were fixed in 10% buffered formalin (Histofix, Carl Roth). They were used to prepare 7 *μ*m slides and stained with hematoxylin (Mayer's hemalum, Merck) and eosin (Eosin G, Carl Roth).

Hematoxylin-eosin (H&E) staining showed that specimens obtained from patients with tumors frequently contained both tumor and remnant nontumorous ductal and exocrine epithelial cells. In contrast to H&E staining, immunofluorescence analysis does not allow the direct discrimination of tumor cells based on the antibody panel used here. Therefore, the immunofluorescence analysis of tissue specimens from tumor patients prior to H&E staining (allocated as “pancreatic cancer” tissue) may partially include nonmalignant transformed epithelial cells of the remnant pancreatic tissue.

### 2.6. Statistical analysis

For statistical analysis, the software SPSS Statistics (version 28.0.0.0, IBM, Armonk, NY, USA) was used. Differences in fluorescence intensity between tumor and normal pancreatic tissues, between different antibody stainings, and differences in tumor growth after treatment were compared using the Mann–Whitney *U*-test. Differences in tumor identification between the macroscopic evaluation and FOM in the CAM model were compared using Wilcoxon's signed rank test. Statistical significance was set at *p* < 0.05.

## 3. Results

### 3.1. Sensitive and Endothelial Cell-Specific Immunofluorescence Imaging of Vital Whole-Mount Tissue

Vital whole-mount human pancreatic tissue was stained with an endothelium-specific antibody (anti-CD31 WM59 mAb) and analyzed using standardized image acquisition. Exposure time, white balance, and gain were set such that highly specific and sensitive visualization of the microvascular system was achieved using both WFM and FOM (Figure 1(c)). Using fluorescence microscopy, normal pancreatic tissue showed a characteristic dense capillary network, whereas pancreatic carcinoma showed an irregular microvascular system of variable density, as visualized by vital whole-mount tissues (Figure 1(c)) and histological slides (Figure 1(c)). The FOM was unable to visualize the fluorescence signal in histological sections.

### 3.2. Immunofluorescence Imaging Using Epithelial Cell-Specific and Anti-CD71 Antibodies

**Figure 2:**
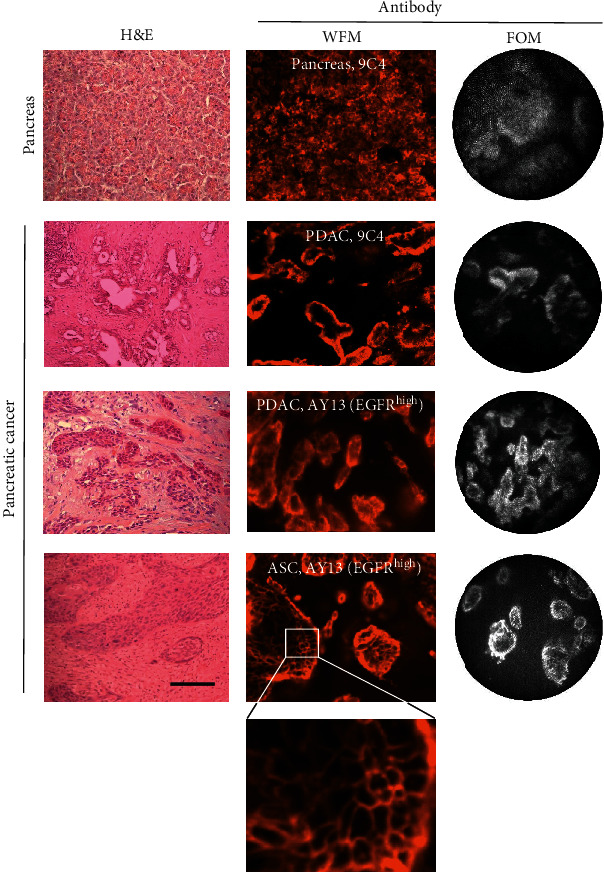
Representative H&E, WFM, and FOM images of vital whole-mount pancreatic tissue samples and tumor tissues. Staining using PE-conjugated anti-EpCAM 9C4 and anti-EGFR AY13 mAbs. Scale bar 200 *μ*m, FOM diameter 790 *μ*m.

Epithelial cell labeling was analyzed in vital whole-mount tissues using the indicated antibody panel (Table 1(b)), whereas isotypic antibodies (Table 1(c)) were used to control the nonspecific binding. Anti-EpCAM 9C4 mAb staining provided a strong and specific signal from morphologically characteristic ductal and exocrine epithelial cells in all analyzed samples of normal and tumor tissues that could be imaged using both WFM and FOM (Figure 2).

**Figure 3:**
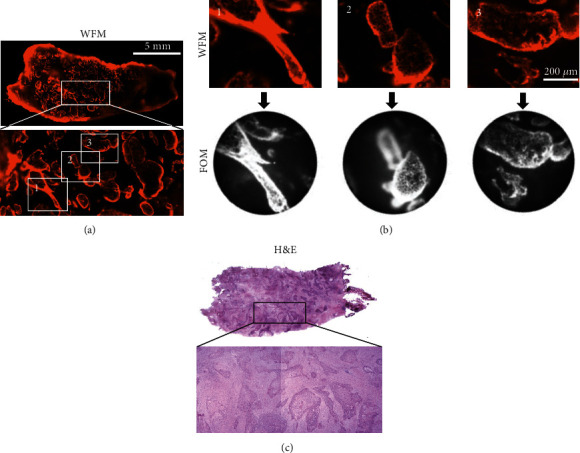
WFM and FOM images of identical regions in vital whole-mount tumor tissue, adenosquamous carcinoma. Staining with anti-EGFR AY13 mAb. (a) Microscopic WFM scan of the whole tissue piece after staining (upper image). The only left site and the middle part of the image contain labeled cell clusters on the dissected surface. Magnification of selected field 2 × 4.5 mm (lower image). (b) Representative imaging of selected WFM and FOM regions. Microscopic images were randomly recorded using FOM and rotated to the axial position of WFM scan to match the WFM image. Identical regions could be well identified both on WFM and FOM and demonstrated comparable quality of WFM and FOM imaging. Representative regions 1-3 on panels (a) and (b) are shown. (c) Microscopic histology image (H&E staining) of the same tissue piece. Scale bars are indicated.

Anti-EGFR AY13 mAb stained cells in normal and tumor tissue samples. The fluorescence intensity was low or moderate in a large majority of samples, and there was no significant difference in MFI between the tumor and normal pancreas (*p* > 0.05). Of the 11 tumor samples, 2 (18%) showed very strong and cell-specific staining (Figures 2 and 3(a)). This produced a high imaging contrast, which was substantially higher than that in normal pancreas and was mainly localized in the cell membrane (Figures 2 and 3(a)). These two tissue samples were classified as EGFR^high^, whereas the other nine samples were classified as EGFR^low^. The EGFR^high^ cellular structures showed identical shapes in immunofluorescence and H&E staining (Figure 2), and it is thus likely that these cells could be identified as malignant tumor cells. One EGFR^high^ sample represented pancreatic ductal adenocarcinoma (PDAC, Figure 2) and another EGFR^high^ tumor represented adenosquamous carcinoma (ASC, Figures 2 and 3). In EGFR^high^ samples, both WFM and FOM produced high imaging contrast, which can be demonstrated by comparison of identical microscopic regions (Figure 3(b)). Interestingly, EGFR^high^ samples were obtained from patients treated with neoadjuvant therapy (FOLFIRINOX). All nine EGFR^low^ samples were obtained from patients who did not receive neoadjuvant treatment.

Labeling with anti-EpCAM CO17-1A mAb showed a substantially lower fluorescence intensity, which was below the detection level for imaging by FOM in the majority of samples (Suppl. Figure 1A). Anti-CK8 1-E8 and anti-CK-18 DA-7 mAb-stained epithelial cells were observed in both normal and tumor tissue samples (Suppl. Figure 1A). However, the fluorescence signal was low and had low specificity, resulting in a low rate of cell visualization using FOM. Anti-CD71 CY1G4 mAb showed absent or very low binding in vital whole-mount tissues. Consequently, visualization of cellular structures in vital whole-mount tissue using this antibody was not feasible.

**Figure 4:**
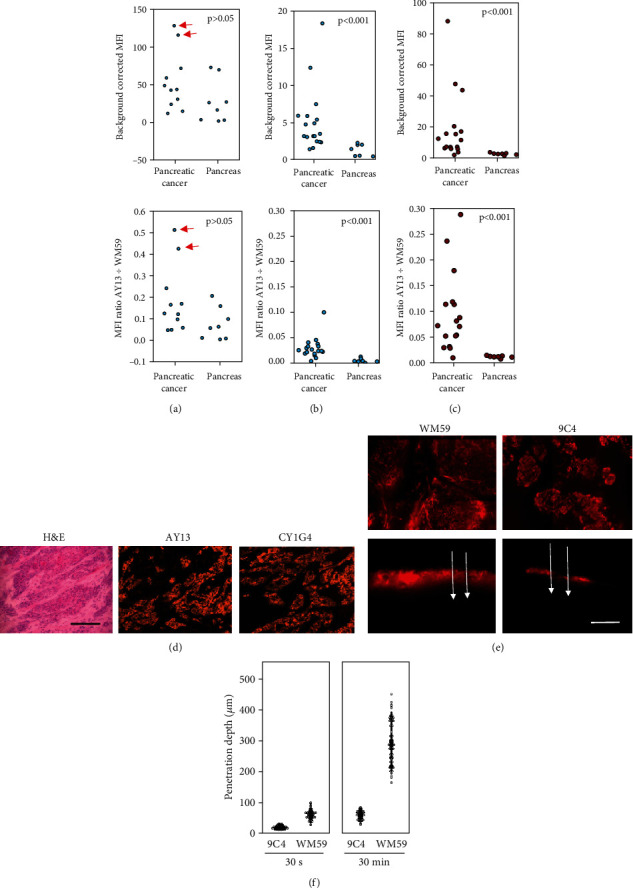
Image-based analysis of the (a) whole-mount tissue and (b) histological slides stained with anti-EGFR AY13 mAb. Background-corrected fluorescence and AY13 ÷ WM59 fluorescence ratio are shown. Red arrows indicate two EGFR^high^ samples. Sample size is given in Table 1(b). (c) Staining of histological slides with anti-CD71 CY1G4 mAbs, image-based analysis of background-corrected fluorescence, and AY13 ÷ WM59 fluorescence ratio and (d) representative images of fluorescence and respective H&E staining of the same tumor tissue sample; scale bar 200 *μ*m. Antibody penetration: (e) representative WFM images of surface tissue (upper images) and cross-sectioned tissue (lower images) (scale bar 1 mm) and (f) quantitative analysis for 30 s (10 *μ*g/mL) and 30 min (2 *μ*g/mL). Data is given as single values measured on 3 independent specimens; for 30 s: *n* = 92 and 87 values for 9C4 and WM59, respectively; for 30 min: *n* = 77 and 132 values for 9C4 and WM59, respectively. Arrows indicate direction of antibody penetration.

In contrast to the vital whole-mount tissue (Figure 4(a) and Suppl. Table 3), anti-EGFR AY13 and anti-CD71 CY1G4 mAbs showed significantly higher specificity in histological slides of tumor tissue samples than in normal pancreas (*p* < 0.001; Figures 4(b), 4(c), and 4(d)). However, the FOM could not image fluorescence on histological slides.

The antibody stained only the tissue surface. The depth of antibody penetration depended on the antibody type and incubation time and varied on average between 60 *μ*m (anti-EpCAM 9C4) and 290 *μ*m (anti-CD31 WM59) after 30 min incubation (Figures 4(e) and 4(f)). Interestingly, the local application of a concentrated antibody solution (10 *μ*g/mL) produced a fluorescence signal sufficient for FOM imaging even after a very short (30 s) incubation (Suppl. Figure 1B).

### 3.3. Microscopic Discrimination of Tumor Tissue Using FOM

The practicability of FOM for tumor discrimination was analyzed using vital tissue labeling with anti-EGFR AY13 or mixed anti-EpCAM 9C4/anti-CD31 WM59 mAbs. Briefly, selected EGFR^high^ tissue specimens (ASC, depicted in Figure 3) and normal tissues were cut into small pieces of 1 mm^3^ (*n* = 21 for tumor, *n* = 14 for normal pancreas) and microscopically evaluated in a double-blind manner. EGFR^high^ tumor cells specifically labeled with anti-EGFR AY13 mAb were well identified using FOM (Suppl. Table 4), whereas discrimination using a combination of epithelial cell patterns and microvascular staining (mixed anti-EpCAM 9C4/anti-CD31 WM-59 mAbs) failed.

### 3.4. FOM of Tumor Cells In Vitro

**Figure 5:**
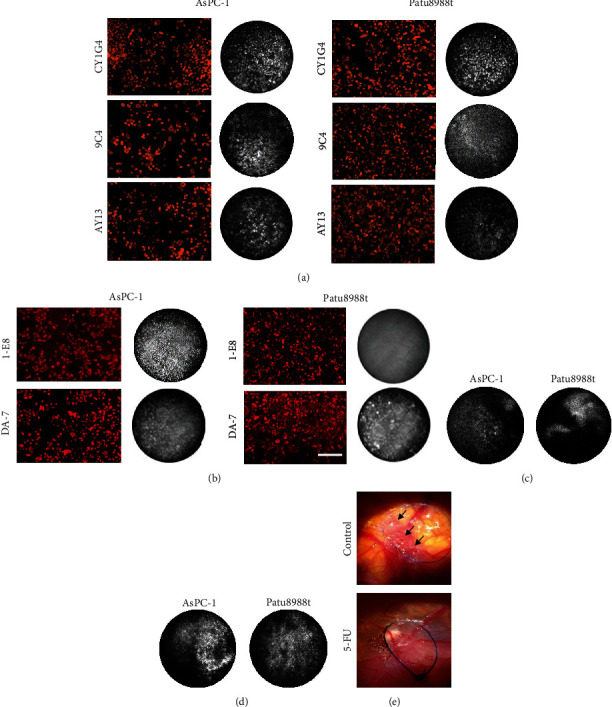
(a) Representative WFM and FOM images of anti-CD71 CY1G4, anti-EpCAM 9C4, and anti-EGFR AY13 mAb binding by vital AsPC-1 and Patu8988t cells in vitro. (b) Representative WFM and FOM images of 1-E8 and DA-7 mAb binding after formalin fixation in vitro; bar 200 *μ*m, FOM diameter 790 *μ*m. Representative FOM images of vital tumor in ovo xenografts stained with PE-conjugated (c) anti-EpCAM 9C4 and (d) anti-EGFR AY13 mAbs. FOM diameter 790 *μ*m. (e) In situ view of tumor growth in CAM model, 8 days after inoculation, control, and 5-FU treatment. The treatment area is additionally marked by blue suture loop. Arrows indicate macroscopic tumor mass.

The usefulness of the FOM for tumor cell imaging in cell culture and in ovo tumor growth was analyzed. In cell cultures, all six examined pancreatic cancer cell lines (AsPC-1, DAN-G, Fampac, MiaPaCa-2, Patu8988t, and Pk-9) showed strong cell membrane binding of anti-CD71 CY1G4 mAb. The binding of anti-EGFR AY13 mAb was strong in 5/6 cell lines, and anti-EpCAM CO17-1A mAb showed low (4/6 cell lines) or moderate (2/6 cell lines) binding (Figure 5(a), Suppl. Table 5, and Suppl. Figure 2). Clones 1E8 and DA-7 bound only after fixation and permeation (Figure 5(b)). Both WFM and FOM provided excellent microscopic visualization of the stained cells in selected cell lines (AsPC, Patu-8988t) (Figures 5(a) and 5(b)). For FOM imaging, fiber optics must directly touch cell monolayer. Although this procedure was performed very carefully, occasional mechanic injury of monolayer and an uneven focus were unavoidable. Therefore, the imaging quality of WFM was mainly better than that of FOM (Figures 5(a) and 5(b)).

### 3.5. Identification of Microscopic Residual Tumors Using FOM In Ovo

All cell lines showed stable progressive growth in ovo with nearly 100% take-on rates. Although the autofluorescence of cell lines was almost absent in cell cultures, it was increased in 4/6 cell lines in ovo; thus, only two non-autofluorescent cell lines (AsPC-1, Patu8988t) were used for further experiments in ovo.

After a short period of tumor growth (2 days after inoculation), staining was achieved after local antibody application. Topic application of concentrated antibody solution produced a sufficient fluorescence signal for FOM imaging after a very short (30-60 s) incubation. Effective tumor-specific labeling was achieved using anti-EpCAM 9C4 (Figure 5(c)) and anti-EGFR AY13 (Figure 5(d)) mAbs. The majority (95%) of microtumors were correctly identified using antibody labeling and FOM (Suppl. Table 6). Intravascular injection of fluorescent AY13 and 9C4 antibodies (1 *μ*g) was not successful for tumor staining and detection using FOM.

Finally, we analyzed the efficacy of FOM for microresidual tumor detection after local chemotherapy. For this analysis, established tumors *in ovo* were locally treated with 5-FU and labeled with a topical antibody as described above. Local chemotherapy led to a significant reduction in macroscopically identifiable vital tumor cell masses (Figure 5(e)). FOM and labeling with PE-conjugated anti-EGFR AY13 (3-day treatment) and anti-EpCAM 9C4 (6-day treatment) mAbs identified additional CAM specimens bearing macroscopically invisible vital tumor microresidues (Table 2).

## 4. Discussion

Fiber-optic microscopy and immunofluorescence were used in our previous study describing microvascular labeling in mouse tumor model [22]. The present work differs fundamentally from the above study and shows the following unique features: (1) the first-time description of immunofluorescence staining using direct immersion of dissected whole-mount tissue into the antibody solution, (2) identification of selected antibody clones suitable for above staining procedure, and (3) use of human tissue. Furthermore, an improved fiber-optic imaging system was designed and constructed for specific aims of the present study.

Immunofluorescence is a basic biomedical technique based on the identification and visualization of fluorescent antibodies that bind to specific antigens. It is mainly used to stain tissue slides or cells in vitro. It has also been used for staining systemically injected or perfused antibodies [22, 30, 31]. In the present study, immunofluorescence staining was performed by labeling of dissected human whole-mount pancreatic tissue which was directly exposed to antibody solution. The application kind resembles the standard immunofluorescence protocols using slides, but tissue fixation and cutting were redundant. Furthermore, the present study was performed on human tissue whereas animal tissue was used in our previous investigations [22].

It was found that antibodies effectively bound to specific antigens localized in the tissue, although the penetration depth was limited. It enables excellent imaging using WFM and provides important morphological insights, such as the presentation of the microvascular system and cellular structures. Interestingly, the antibody-binding properties of vital whole-mount tissue and histological slides were different. For example, anti-CD71 CY1G4 mAb stained epithelial cells in histological slides of tumor tissue samples and vital cells in vitro, but not in whole-mount tissue. These discrepancies could result from sectioning and fixation, which change antibody binding and penetration in tissue slides. In particular, accessibility to cellular epitopes may be an important factor [32, 33] that differs between whole-mount and sectioned tissues. Furthermore, the tissue thickness that produces fluorescence in histological slides was limited to 7 *μ*m. The fluorescence signal in whole-mount tissue imaging is determined by the penetration of the light and antibodies. As shown in the present study, the penetration of the anti-EpCAM 9C4 mAb after 30 min of incubation (approximately 60 *μ*m) was substantially higher than the thickness of histological slides. It can also be proposed that anti-CD31 WM59 mAb freely diffuses into the lumen of the microvascular system and binds to the endothelium, showing even greater penetration (approximately 200-400 *μ*m) than anti-EpCAM 9C4 mAb. Wide-field fluorescence microscopy collects and focuses light from the superficial tissue layers. Although the thickness of this layer is dependent on local light absorption, it is substantially higher than the thickness of the histological slides and substantially lower than the depth of antibody penetration.

Fluorescence staining was detectable using FOM and provided imaging of sufficient contrast and quality. FOM detected only strongly stained structures and achieved a visibility level in tissue samples with sufficient antigen density using anti-EpCAM 9C4, anti-EGFR AY13, and anti-CD31 WM59 mAbs. The lower sensitivity can be explained by the loss of light energy in the fiber optics [34]. Furthermore, the resolution of the FOM is limited by the size of the single fibers. In our setting, it provided microscopic imaging of 790 *μ*m in diameter, comparable to the WFM imaging dimensions, using a 10× objective (600 × 800 *μ*m). In the present study, FOM provided excellent microscopic imaging of vital cells in whole-mount human tissues, cell monolayers in vitro, and solid tumor growth in ovo.

In the present study, only anti-EGFR AY13 mAb showed a very specific signal in whole-mount tumors (identified as EGFR^high^) treated with neoadjuvant chemotherapy. Despite these interesting results, the sample size analyzed in this study was too small, and further studies are needed to clarify the relationship between neoadjuvant chemotherapy and anti-EGFR-based tumor detection. In the present study, we used one of these EGFR^high^ samples and sample partitions containing tumors to study their potential for microtumor identification. These pilot data indicate an observable high signal intensity that enables blind and precise discrimination of malignant microresidues and/or remnant nonmalignant pancreatic epithelial cells using FOM.

We identified a third antibody clone, anti-EpCAM 9C4 mAb, which showed high signal intensity by binding to cellular structures in vital whole-mount tissues. Binding of anti-EpCAM 9C4 mAb yielded an excellent signal-to-noise ratio for FOM, by which the characteristic cellular structures formed by epithelial cells could be imaged. Based on the use of a general epithelial marker for cell detection, the discrimination between normal and malignant tissues based on blind identification of cellular morphology failed.

As mentioned above, the analyzed tumor tissue samples frequently contained both tumor and remnant nonneoplastic pancreatic tissues. Such a mixed malignant/nonmalignant morphology could reflect the characteristics of the peripheral invasive tumor margin. Malignant tumor cells can be confidently identified using conventional H&E staining, and the use of the markers in this study combined with immunofluorescent microscopy was inferior to common diagnostic workflows in pathology. The anti-EGFR AY13 mAb could represent a tool for the specific detection of EGFR^high^ tumors, especially when combined with more tumor-specific antibodies in a simultaneous multicolor codetection approach, but this requires further investigation.

As was also demonstrated in the present study, a very short exposure time of the antibody to the antigen was achieved using a topical antibody application by a minibrush. This was sufficient to stain the tissue surface and enable FOM imaging of whole-mount human tissue. This procedure was also used to stain vital tumors in ovo and identify tumor microresidues that persisted after local chemotherapy. The FOM was effective in finding microscopic tumor remnants in ovo that were not macroscopically visible. This result additionally substantiates the potential of FOM for identifying intraoperative *in situ* tumor microresidues.

The short exposure time of antibodies to antigens may be particularly important in situations with a limited time window. One example of such a situation is the direct identification of microresidues in the dissection margin during resection (R1 resection). This is important for surgeries involving cancer types with high R1 resection rates, such as pancreatic [35] and liver [36] cancers. We are aware that this concept is currently unsustainable. Although FOM imaging in combination with topical antibody application may represent a practical tool, antibodies/markers with high tumor specificity must be identified to realize this concept.

Last decade, other methods for intraoperative in situ detection of tumors and tumor margins were suggested. These methods are mainly based on mass spectroscopy but use different principles of local tissue extraction [37, 38]. Pilot clinical studies showed usefulness of these techniques for detection of pancreatic [39], breast [40], and thyroid [41] cancer. Further studies should compare FOM imaging and other methods.

## 5. Conclusion

In summary, a combination of vital tissue immunofluorescence staining and fiber-optic microscopy could be a useful technology for discriminating residual tumor mass in pancreatic tissue *in situ*. For potential clinical applications, antigens/antibodies with high specificity for tumor cells must be identified.

## Figures and Tables

**(a):** 

Frozen tissue/histological slides	
Total	19
Age	60 ± 6
Sex (M/F)	8/11
G (1/2/3)	0/9/10
Neoadjuvant treatment (yes/no)	0/19
Fresh whole-mount tissue	
Total	24
Age	64 ± 8
Sex (M/F)	16/8
G (1/2/3/unknown)	0/14/7/3
Neoadjuvant treatment (yes/no)	3/21

**(b):** 

Antigen	MAb clone	Isotype	Catalog ID (all from BioLegend)			Number of samples	
			PE-conjugated	AF647-conjugated	Purified	Pancreas	Pancreatic cancer
CD326/EpCAM	CO17-1A	IgG2a	369805			11	18
CD326/EpCAM	9C4	IgG2b	324205	324212		13	15
CD71/TFRC	CY1G4	IgG2a	334106	334118		8	11
CD31/PECAM	WM59	IgG1	303112	303106		18	19
EGFR	AY13	IgG1	352904	352918		8	11
CK18	DA-7	IgG1			628402	3	7
CK8	1-E8	IgG2a			904804	3	7

**(c):** 

Antigen	MAb clone	Isotype	Catalog ID (all from BioLegend, if not otherwise indicated)		
			PE-conjugated	AF647-conjugated	Purified
Mouse IgG1	MOPC-21	IgG1		400130	
Mouse IgG1	P3.6.2.8.1	IgG1	12-4714-41 (Thermo)		
Mouse IgG2a	MOPC-173	IgG2a		400234	
Mouse IgG2b	MG2b-57	IgG2b			401201

**Table 2:** Identification of vital tumor residues using only macroscopic and combined macroscopic and FOM inspection after topical 5-FU chemotherapy for 3 and 6 days in ovo tumor model.

3 days	Macroscopic	Macroscopic+FOM
PaTu8988t (*n* = 7)	1 (14%)	4 (57%)
AsPc-1 (*n* = 5)	2 (40%)	3 (60%)
6 days	Macroscopic	Macroscopic+FOM
PaTu8988t (*n* = 22)	4 (18%)	10 (45%)
AsPc-1 (*n* = 22)	3 (14%)	9 (41%)

## Data Availability

Original data can be made available upon reasonable request.

## Abbreviations

FOM: Fiber-optic microscopy
WFM: Wide-field microscopy
5-FU: 5-Fluouracil
CAM: Chorioallantoic membrane
EGFR: Epidermal growth factor receptor
mAb: Monoclonal antibody.

## Supplementary Materials

Supplementary Materials Supplementary Figure 1: (A) representative WFM and FOM images of vital whole-mount pancreatic and tumor tissue, stained using Alexa Fluor 647-conjugated anti-CK8 1-E8, anti-CK-18 DA-7, and PE-conjugated anti-EpCAM CO17-1A mAbs. Coupled WFM-FOM images represent the same tissue sample; bar 200 *μ*m, FOM diameter 790 *μ*m. (B) FOM imaging of whole-mount pancreatic cancer tissue after short exposure to PE- or Alexa Fluor 647-conjugated anti-EpCAM 9C4 antibody; scale bar 200 *µ*m, FOM diameter 790 *μ*m. Supplementary Figure 2: representative WFM and FOM images of pancreatic cancer cell lines in vitro; scale bar 200 *µ*m, FOM diameter 790 *μ*m. Supplementary Table 1: specifications of the fluorescence detection using WFM. Supplementary Table 2: specifications of the fluorescence detection using FOM. Supplementary Table 3: row and background-corrected MFI values of PE-conjugated anti-EGFR AY13 and Alexa Fluor 647-conjugated anti-WM59 antibodies. Supplementary Table 4: double-blind discrimination of tumor tissue (A) and calculation of false and correct results (B) in selected EGFR^high^ tissue sample (ASC) using FOM; data are given as number (percentage) of tissue pieces (1 mm^3^). Supplementary Table 5: intensity of the vital tumor cell staining *in vitro.* MAb staining: ++ strong, + moderate, and − no/poor. Supplementary Table 6: double-blind discrimination of microtumors in CAM (A) and calculation of false and correct results (B) using FOM; data are given as number (and percentage) of eggs.
