# Supplementary material for: In Situ Immunofluorescence Imaging of Vital Human Pancreatic Tissue Using Fiber-Optic Microscopy
Source: Int J Biomed Imaging. 2024 Jun 6;2024:1397875. doi: 10.1155/2024/1397875 (PMC11178408; doi:10.1155/2024/1397875)
Supplement: Supplementary Materials — Supplementary Figure 1: (A) representative WFM and FOM images of vital whole-mount pancreatic and tumor tissue, stained using Alexa Fluor 647-conjugated anti-CK8 1-E8, anti-CK-18 DA-7, and PE-conjugated anti-EpCAM CO17-1A mAbs. Coupled WFM-FOM images represent the same tissue sample; bar 200 μm, FOM diameter 790 μm. (B) FOM imaging of whole-mount pancreatic cancer tissue after short exposure to PE- or Alexa Fluor 647-conjugated anti-EpCAM 9C4 antibody; scale bar 200 µm, FOM diameter 790 μm. Supplementary Figure 2: representative WFM and FOM images of pancreatic cancer cell lines in vitro; scale bar 200 µm, FOM diameter 790 μm. Supplementary Table 1: specifications of the fluorescence detection using WFM. Supplementary Table 2: specifications of the fluorescence detection using FOM. Supplementary Table 3: row and background-corrected MFI values of PE-conjugated anti-EGFR AY13 and Alexa Fluor 647-conjugated anti-WM59 antibodies. Supplementary Table 4: double-blind discrimination of tumor tissue (A) and calculation of false and correct results (B) in selected EGFRhigh tissue sample (ASC) using FOM; data are given as number (percentage) of tissue pieces (1 mm3). Supplementary Table 5: intensity of the vital tumor cell staining in vitro. MAb staining: ++ strong, + moderate, and − no/poor. Supplementary Table 6: double-blind discrimination of microtumors in CAM (A) and calculation of false and correct results (B) using FOM; data are given as number (and percentage) of eggs. [file 1397875.f1.docx]

Supplementary Table 1.

| Fluorophore | Light source, LED (Zeiss) | Light power apllied to the subject | Excitation, nm | Beam splitter, nm | Emission, nm |
| --- | --- | --- | --- | --- | --- |
| DAPI, Hoechst33342 | 385 nm | 10 mW | 370-400 | 405 | 410-440 |
| Phycoerythrin | 555 nm | 5.3 mW | 540-570 | 575 | 580-605 |
| AlexaFluor 647 | 625 nm | 41 mW | 615-648 | 653 | 659-759 |

Supplementary Table 2.

| Fluorophore | Light source, LED (Thorlabs) | Light power applied to the subject | Excitation, nm | Beam splitter, nm | Emission, nm |
| --- | --- | --- | --- | --- | --- |
| Phycoerythrin | MINTL | 0.20 mW | 525-565 | 582 | 570-590 |
| AlexaFluor 647 | M660L4 | 0.34 mW | 590-670 | 669 | 670-690 |

Supplementary Table 3.

| Clone | AY13 | | WM59 | |
| --- | --- | --- | --- | --- |
| Sample ID | row | background-corrected | row | background-corrected |
| 1 | 220.10 | 129.78 | 513.89 | 250.23 |
| 2 | 226.52 | 113.78 | 446.69 | 271.55 |
| 3 | 148.64 | 41.52 | 634.29 | 367.45 |
| 4 | 125.12 | 17.28 | 625.43 | 334.73 |
| 5 | 152.40 | 70.00 | 629.13 | 407.75 |
| 6 | 130.06 | 44.68 | 540.89 | 279.39 |
| 7 | 126.80 | 33.48 | 479.96 | 238.66 |
| 8 | 133.64 | 59.84 | 435.79 | 243.59 |
| 9 | 137.46 | 11.44 | 615.95 | 232.89 |
| 10 | 143.34 | 50.40 | 644.12 | 409.58 |
| 11 | 112.58 | 24.46 | 690.73 | 409.57 |

Supplementary Table 4.

|  | total | correct discrimination | failed discrimination |
| --- | --- | --- | --- |
| tumor | 16 | 14 | 2 |
| normal pancreas | 21 | 21 | 0 |

|  | negative | positive |
| --- | --- | --- |
| false | 2 (9%) | 0 (0%) |
| correct | 21 (100%) | 14 (91%) |

Supplementary Table 5.

| Cell line | CY1G4 | CO17-1A | AY13 |
| --- | --- | --- | --- |
| AsPC-1 | ++ | + | ++ |
| DAN-G | ++ | + | ++ |
| Fampac | ++ | - | - |
| MiaPaCa-2 | ++ | - | ++ |
| Patu-8988t | ++ | - | ++ |
| Pk-9 | ++ | - | ++ |

Supplementary Table 6.

|  |  | correct discrimination | failed discrimination |
| --- | --- | --- | --- |
| tumor | 75 | 71 | 4 |
| control (normal CAM) | 37 | 34 | 3 |

|  | negative | positive |
| --- | --- | --- |
| false | 3 (8%) | 4 (5%) |
| correct | 34 (92%) | 71 (95%) |

Supplementary Figure 1

9C4-PE

9C4-AF647

9C4

CO17-1A

DA-7

1-E8

A

Pancreas

WFM

FOM

B

Pancreatic cancer

WFM

FOM

Supplementary Figure 2

autofluorescence

**MiaPaCa-2**

CY1G4

AY13

CO17-1A

**DAN-G**

**Fampac**

**Pk-9**

autofluorescence

CY1G4

AY13

CO17-1A

autofluorescence

CY1G4

AY13

CO17-1A

autofluorescence

CY1G4

AY13

CO17-1A

**FOM**

**WFM**

**FOM**

**WFM**

**FOM**

**WFM**

**FOM**

**WFM**
